# Sucrose Utilization in Budding Yeast as a Model for the Origin of Undifferentiated Multicellularity

**DOI:** 10.1371/journal.pbio.1001122

**Published:** 2011-08-09

**Authors:** John H. Koschwanez, Kevin R. Foster, Andrew W. Murray

**Affiliations:** 1 FAS Center for Systems Biology, Harvard University, Cambridge, Massachusetts, United States of America; 2 Department of Molecular and Cellular Biology, Harvard University, Cambridge, Massachusetts, United States of America; 3 Department of Zoology, University of Oxford, Oxford, United Kingdom; 4 Oxford Center for Integrative Systems Biology, University of Oxford, Oxford, United Kingdom; University of Lausanne, Switzerland

## Abstract

We use the budding yeast, *Saccharomyces cerevisiae*, to investigate one model for the initial emergence of multicellularity: the formation of multicellular aggregates as a result of incomplete cell separation. We combine simulations with experiments to show how the use of secreted public goods favors the formation of multicellular aggregates. Yeast cells can cooperate by secreting invertase, an enzyme that digests sucrose into monosaccharides, and many wild isolates are multicellular because cell walls remain attached to each other after the cells divide. We manipulate invertase secretion and cell attachment, and show that multicellular clumps have two advantages over single cells: they grow under conditions where single cells cannot and they compete better against cheaters, cells that do not make invertase. We propose that the prior use of public goods led to selection for the incomplete cell separation that first produced multicellularity.

## Author Summary

The evolution of multicellularity is one of the major steps in the history of life and has occurred many times independently. Despite this, we do not understand how and why single-celled organisms first joined together to form multicellular clumps of cells. Here, we show that clumps of cells can cooperate, using secreted enzymes, to collect food from the environment. In nature, the budding yeast *Saccharomyces cerevisiae* grows as multicellular clumps and secretes invertase, an enzyme that breaks down sucrose into smaller sugars (glucose and fructose) that cells can import. We genetically manipulate both clumping and secretion to show that multicellular clumps of cells can grow when sucrose is scarce, whereas single cells cannot. In addition, we find that clumps of cells have an advantage when competing against “cheating” cells that import sugars but do not make invertase. Since the evolution of secreted enzymes predates the origin of multicellularity, we argue that the social benefits conferred by secreted enzymes were the driving force for the evolution of cell clumps that were the first, primitive form of multicellular life.

## Introduction

During evolution, smaller and simpler elements have repeatedly come together to make bigger and more complicated functional units; examples include genes forming genomes and individuals forming societies. Multicellular organisms are societies of cells and the transition from single to multicelled groups arises in two ways [1],[2]: (1) single cells come together to form groups that subsequently differentiate into different cell types (e.g., slime molds and myxobacteria), or (2) the offspring of a single cell stay stuck together after cell division. This second mode—incomplete cell separation—appears to be a critical step in the independent origins of multicellularity that led to animals, plants, and colonial algae [3]. However, the origins of incomplete cell separation are obscure: the ancestors of current multicellular organisms are ancient and the interpretation of early multicellular fossils [4],[5] remains a challenge [6].

Despite these difficulties, taxonomic groups that contain both multicellular and unicellular species have provided insights into the origin of multicellularity. The Volvocaceae are a family of algae that range from single celled species through undifferentiated groups of cells to species with differentiated germ line and somatic cells. In this group multicellularity appears to have arisen through a series of stages with incomplete cell separation occurring early on in the transition [7]. The choanoflagellates, which are related to basal animals such as sponges, exist in both single celled and colonial forms, and also form colonies through incomplete cell division [8]. We focus on what was likely to be the initial step in the evolution of multicellularity, the appearance of aggregates of undifferentiated groups of cells, and ignore two crucial later stages common to plants and animals: the division of labor between different cell types and reproduction through single-celled propagules.

We used the genetic tractability of the budding yeast, *Saccharomyces cerevisiae*, to study the simplest form of multicellularity: an undifferentiated group of cells that remain attached to each other after cell division. Our goal was to find conditions where cells that remain attached to one another have an advantage over isolated cells. We genetically manipulated two traits of budding yeast. The first is cell separation. After cytokinesis, the physical separation of the two daughter cells requires digestion of part of the cell wall [9]. Many natural isolates of *S. cerevisiae* show incomplete separation and form clumps, whereas laboratory strains have been selected to show complete separation and exist as isolated cells [10]. The second is the secretion of hydrolytic enzymes that act on more complex molecules to release nutrients, which act as public goods that cells can take up. Enzyme secretion is a form of cooperation because the nutrients the enzymes release can increase the fitness of cells other than the secreting cell. Yeast secrete a number of enzymes, including acid phosphatase (Pho5) [11], phospholipase (Plb2) [12], and invertase (Suc2) [13], that release nutrients from molecules in the medium. Here we focus on invertase.

Invertase breaks down the disaccharide sucrose into the monosaccharides glucose and fructose. The secretion of invertase from budding yeast has long been studied. In the 19^th^ century, Berthelot and Pasteur quarreled over the mechanism responsible for the invertase action [14] and Fischer's studies of invertase in the early 20^th^ century led to the “lock and key” concept of enzyme specificity [15]. More recently, key aspects of glucose repression and protein secretion were discovered by studying invertase [16]–[22], and invertase secretion has served as a model for studies of cooperation among budding yeast [23],[24].

Here we explore the interaction between incomplete cell separation and the use of invertase as a secreted product that promotes the growth of neighboring cells. Our goal was to ask if cooperative enzyme secretion and the formation of groups of genetically identical cells could have led to the origin of multicellular life. Our data suggest that the use of secreted products can indeed lead to natural selection for incomplete cell separation.

## Results

### Lab Yeast Cannot Grow from a Single Cell in Low Concentrations of Sucrose

**Figure 1:**
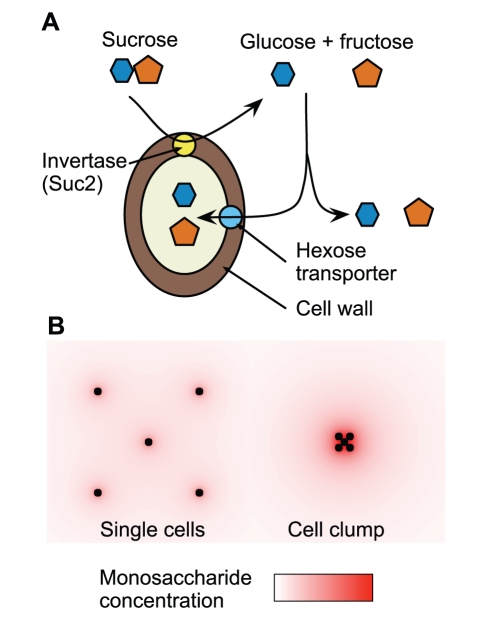
Extracellular hydrolysis of sucrose allows other cells to share glucose and fructose. (A) Sucrose is hydrolyzed into glucose and fructose by invertase located in the cell wall. The glucose and fructose are imported into the cell by hexose transporters or escape into the medium by diffusion. (B) The glucose and fructose monosaccharides diffuse away from the cell wall and are more easily shared between cells when the cells are clustered in a clump (right) than when the cells are spaced apart (left).

We began by characterizing the growth of single yeast cells in medium with sucrose as the only carbon source, an environment that requires invertase secretion to allow cell proliferation. At low glucose concentrations, invertase, encoded by the *SUC2* gene, is secreted in a glycosylated, octameric form (Figure S1) [25],[26]. The invertase octamer is retained in the cell wall, where it hydrolyzes the sucrose in the media into glucose and fructose. After hydrolysis, each glucose and fructose molecule either diffuses away from the cell or is captured by sugar transporters in the cell membrane (Figure 1A). The sugar influx into the cell therefore depends on the rates of sucrose diffusion to the cell wall, sucrose hydrolysis at the cell wall, and capture of the diffusing monosaccharides at the cell membrane. Contrast this with the case of a cell grown in glucose and fructose, where the sugar flux into the cell depends only on the rate of monosaccharide diffusion and capture at the cell membrane. If three conditions are satisfied, there should be a sugar concentration that allows growth on glucose and fructose but not on sucrose: (1) the net monosaccharide flux into a cell grown in sucrose is less than the monosaccharide flux of a cell grown in equivalent molarity glucose and fructose, (2) there is minimum monosaccharide flux required for growth, and (3) there is no sucrose import into the cell. In addition, the threshold concentration for growth on sucrose should depend on cell density because some of the monosaccharides that escape from one cell can be captured by its neighbors.

**Figure 2:**
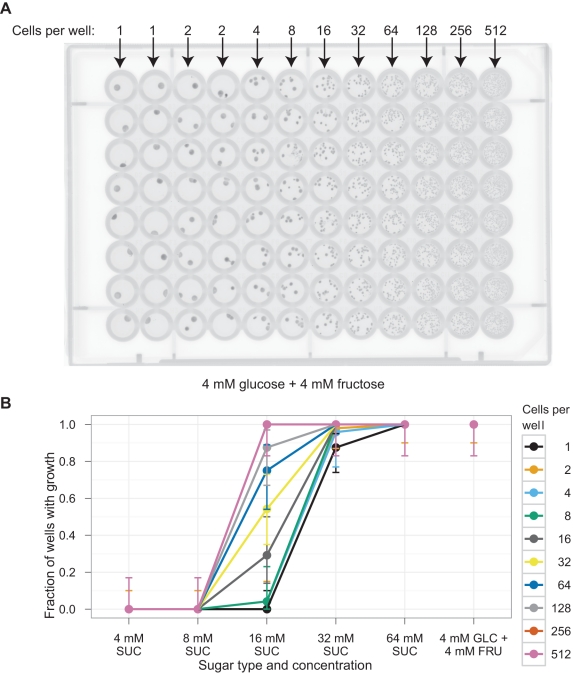
Lab yeast strains, labeled with YFP, cannot grow at low sucrose concentrations. Cells were sorted into microtiter wells, each containing 150 µl of medium, at the given cell density and sugar concentration and allowed to grow for 85 h without shaking at 30°C. (A) Typical fluorescent scan of a plate containing 4 mM glucose plus 4 mM fructose inoculated with the indicated number of cells. (B) The fraction of wells with growth using strain yJHK111 (W303 genetic background). Results shown are totals of three experiments; each experiment used one plate per sugar concentration. Error bars refer to 95% binomial confidence interval using the adjusted Wald method. See Figure S2 for results using a strain with the S288C background.

To test these predictions, we used a fluorescence activated cell sorter (FACS) to inoculate between 1 and 512 single budding yeast cells of a standard laboratory strain background (W303) into each well of a 96-well microtiter plate. Each well contained 150 µl of media that contained one of two carbon sources: sucrose or a mixture of glucose and fructose. The plates were examined after being left stationary at 30°C for 85 h. Figure 2A shows that each cell placed into medium containing 4 mM glucose plus 4 mM fructose formed a visible microcolony, whereas Figure 2B shows that even at 8 mM sucrose (equivalent to 8 mM glucose plus 8 mM fructose), inoculating as many as 512 single cells per well failed to lead to visible growth. Growth at 16 mM sucrose was cell density dependent: very few of the wells inoculated with a single cell produced visible growth, but there was growth in every well inoculated with 512 cells. Figures 2B and S2 show that two different strain backgrounds, W303 and S288C, gave similar results. (All strains in this study are prototrophic and constitutively express a fluorescent protein to allow FACS selection and fluorescence-based imaging.) The results in Figures 2B and S2 cannot be explained by cells making a stochastic decision whether to proliferate or not in sucrose. For this to be the case, a small percentage of cells would have grown regardless of cell density. Instead, we see that in wells where growth occur, the number of microcolonies is roughly equal to the number of cells deposited (Figure S3).

Observing density-dependent growth at 16 mM sucrose leads to two conclusions. The first is that an individual cell fails to capture much of the glucose and fructose produced by the invertase located in its cell wall. If cells could capture all the monosaccharides they produced, growth would not be density-dependent. The second is that there is a group benefit to inoculation in sucrose: each cell can benefit from the monosaccharides that its neighbors produced but failed to capture. Because this benefit depends on diffusion, it should be greatest if the cooperating cells are touching each other.

### Cell Clumps Grow in Sucrose Concentrations Where Single Cells Cannot

If a clump of yeast cells (rather than a single yeast cell) is inoculated into sucrose media, the hydrolyzed fructose and glucose will still diffuse away from each cell wall. But each cell can take up some of the monosaccharides that escape from its neighbors. This effect is strongest at the center of the clump and could raise the rate of sugar influx to a level that would allow clumps of cells to grow in sucrose concentrations that are too low to allow the proliferation of single, isolated cells (Figure 1B).

We explored this scenario by simulating sugar hydrolysis, diffusion, and import by single cells and clumps. The simulation neglects cellular growth during the simulation time and assumes that all diffusion is radial and that glucose and fructose are taken up with the same rates. The simulation starts with a single cell in the center of a 150 µl spherical volume that mimics a microtiter well. All the parameters used in the simulation come from our measurements or those in the literature (Table S1). At each time interval, the simulation performs the following steps: (1) the monosaccharide concentration at the cell membrane is measured to determine the rates of invertase production and monosaccharide import; (2) invertase is produced and secreted to the cell wall; (3) sucrose is hydrolyzed into monosaccharides; (4) monosaccharides are imported into the cell; and (5) sucrose and monosaccharides are allowed to diffuse through the media. We compared two arrangements of 30 cells: either as a single clump of cells immediately surrounding the center cell or dispersed as individual cells throughout the well. To simulate the geometry of cells surrounding the central cell of a clump, a “mean field” of cellular mass is assumed to surround the cell. Diffusion is solved using the Crank-Nicolson method [27]. Details of the software and all parameters used are described in Table S1.

**Figure 3:**
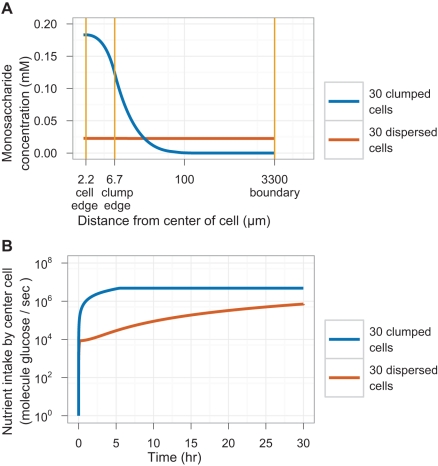
Simulation of glucose uptake in isolated cells and a cell clump. The simulated local glucose concentration and glucose uptake of a cell inoculated at the center of a 150 µl sphere of 8 mM sucrose in two environments: at the center of a clump of 30 cells and at the center of a total of 30 cells uniformly dispersed throughout the volume. (A) Glucose concentration as a function of radial distance from the center of the cell after 30 h of incubation. Note the logarithmic scale on the *x*-axis. (B) Glucose intake rate of the cell as a function of time after inoculation. Note the logarithmic scale on the *y*-axis. See Supporting Information for details of code and parameters.

Figure 3 shows the simulated glucose concentrations and uptake rates 30 h after inoculating 30 single cells or a single clump of 30 cells. The cell at the center of the clump reaches an equilibrium intake of ≈5E6 glucose molecules/s, while an isolated cell reaches an intake rate of only ≈7E5 glucose molecules/s after 30 h (although this value will continue to rise as the sucrose in the well is hydrolyzed). Cells dividing very slowly at very low glucose concentrations in chemostats have a measured intake rate of ≈1-2E6 glucose molecules/s [28],[29], arguing that this is approximately the minimum glucose uptake rate to support cell proliferation. As a result, we predicted that single cells will not grow at 8 mM sucrose, whereas clumps of cells will. We repeated this simulation for 2 mM sucrose and 32 mM sucrose: the simulation predicts that clumps reach equilibrium values of ≈1E6 and ≈1E7 glucose molecules/s, while isolated cells reach glucose intake rates of ≈3E5 and ≈1E6 glucose molecules/s after 30 h (Figure S4). We also examined the effect of initial clump size on glucose intake of the center cell by repeating the simulation in 8 mM sucrose over 8 h for different clump sizes (Figure S5). The monosaccharide concentration and intake rate of the central cell peak at a clump radius of ≈30 µm, corresponding to roughly 1,000 cells, after which the cells in the center begin to starve for nutrients.

**Figure 4:**
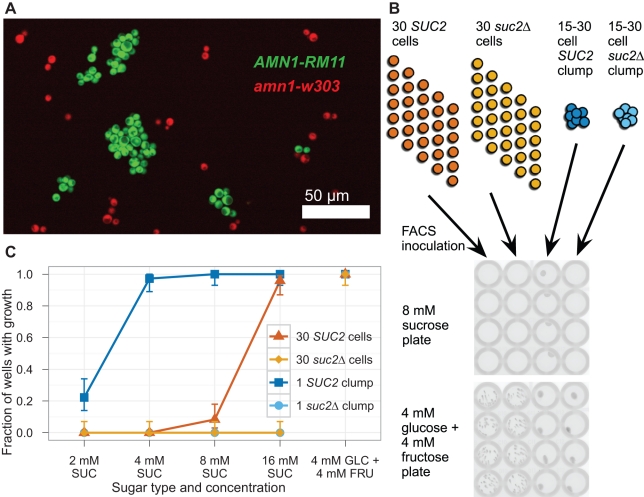
Clumps of cells have a growth advantage over an equal number of single cells in low sucrose concentrations. (A) A 40× confocal image of W303 yeast cells showing the clumpiness phenotype of two different alleles of *AMN1*. The *amn1-W303* (non-clumpy) strain yJHK112 constitutively expressed mCherry driven by the *ACT1* promoter and is shown in red. The *AMN1-RM11* (clumpy) strain yJHK223 constitutively expressed mCitrine driven by the *ACT1* promoter and is shown in green. (B) FACS inoculation. All cells expressed mCitrine driven by the *ACT1* promoter. By gating on pulse width and fluorescence, clumps of 15 to 30 cells were differentiated from single cells. The number of cells and the gating for each well was set on the FACS software. After 85 h of growth without shaking at 30°C, plates were read on a fluorescent scanner and growth was scored by manually counting colonies. (C) Comparison of growth between inoculations of clumps of 15–30 cells and 30 individual cells using clumpy, *AMN1-RM11* strains yJHK223 (*SUC2*) and yJHK224 (*suc2*Δ). Results shown are totals of three experiments; each experiment used one plate for each sugar concentration, which represents 24 wells for each combination of genotype and clumpiness. Error bars refer to 95% binomial confidence interval using adjusted Wald method. The *suc2-1cyt* (cytoplasmic-invertase only) strain yJHK259 was also tested and did not grow in any well in 2, 4, 8, or 16 mM sucrose media and grew in 100% of the wells in 4 mM glucose+4 mM fructose media (not shown).

We made strains to experimentally test the prediction that clumps could grow at low sucrose concentrations but isolated cells could not. Haploid cells from the vineyard isolate strain RM11 fail to fully separate their cell walls after cytokinesis and grow in clumps rather than single cells, whereas the lab strains exist mostly as single cells. Kruglyak and colleagues showed that the genetic difference responsible for the difference in cell wall separation lies in the *AMN1* gene [30]. If *amn1-W303*, the *AMN1* allele from lab yeast, is replaced by *AMN1-RM11*, the allele from the wild yeast strain RM11, lab strains acquire the clumpy phenotype of wild yeast (Figure 4A).

We sorted single cells from clumps to compare their ability to grow in low sucrose concentrations. We used FACS to inoculate 30 single cells and single clumps of 15–30 cells of an *AMN1-RM11* strain in alternating wells of a 96 well plate, as shown in Figure 4B. The plates were kept stationary at 30°C for 85 h and then scanned on a fluorescent scanner. Figure 4C shows that in all three strains, only cells inoculated as a clump could grow in a majority of the wells containing 4 mM and 8 mM sucrose. This growth was dependent on the production and secretion of invertase. Cells that lacked the invertase gene (*suc2Δ*) could not grow as single cells or clumps; nor could cells that produced invertase but failed to secrete it (*suc2-1cyt*). Our results confirm the prediction that a clump of cells can grow in sucrose concentrations where an equivalent number of single cells cannot.

Because Amn1 affects the expression of many genes and Amn1 is expressed during growth in the well, we used two other methods of producing multicellular aggregates to confirm the advantage of clumps over single cells. The first was to place *AMN1* under the control of the conditional *GAL1* promoter, which allowed us to manipulate the clumpiness of cells before they were exposed to sucrose, but ensured that both clumps and single cells expressed a low level of Amn1 during the growth assays (since the wells did not contain galactose). The second was to conditionally express *CTS1*, whose gene product, chitinase, is responsible for degrading the primary septum between mother and daughter yeast cell. When *CTS1* expression is controlled by the *GAL1* promoter, cultures grown with galactose contain single cells, and cultures grown without galactose contain clumps. Both methods produced the same results: clumps grew at sucrose concentrations where single cells could not (Figure S6).

The increase in monosaccharide concentration in the clump could give clumps two advantages over a single cell. The first is that more monosaccharide is available for consumption in clumps because cells capture more of the monosaccharides produced by their neighbors, as explained above. The second depends on the fact that low levels of glucose induce invertase expression as reported by Dodyk and Rothstein [13] and confirmed in Figure S1A. This regulation creates a positive feedback loop: higher glucose levels in the clump will lead to higher levels of invertase production, which will lead to higher glucose levels until the glucose concentration reaches a value of approximately 0.25–1 mM, above which additional glucose represses invertase expression.

To show that the advantage of clumps is not solely dependent on this positive feedback loop we manipulated cells to give us control of invertase expression and repeated the comparison of clumps and single cells. We placed the *SUC2* gene under the control of the galactose-inducible *GAL1* promoter and deleted the *GAL1* and *GAL10* genes so that cells could only use galactose to control gene expression and not as a carbon source [31]. At three different, low levels of galactose induction, clumps of yeast grew at levels at which an equivalent number of single cells did not grow (Figure S7). Because we had broken the positive feedback loop between the glucose concentration and invertase expression, we conclude that the advantage of inoculation as a clump is not solely due to regulation of *SUC2*.

Simulation predicts and experiment confirms that a clump of yeast cells have a growth advantage over single yeast cells in low concentrations of sucrose. This advantage is due to the increased levels of glucose in the center of the clump available for both regulation of invertase expression and for glucose consumption. Below, we discuss the significance for the early evolution of multicellular life.

### Competition against *suc2Δ* Cells

Because invertase is a secreted public good, it has been used to investigate social interactions amongst microbes [23],[24]. Cells that cannot make invertase are often referred to as “cheaters” since they can grow on the monosaccharides that are liberated when invertase-producing cells hydrolyze sucrose. Cells that produce invertase incur a fitness cost, which we measured to be 0.35% for cells that are forced to express invertase and grown in 1 mM glucose (Table S3). When a mixture of *suc2Δ* and *SUC2* cells are inoculated together on plates, their fate depends on their density. At low densities, the ratio of *SUC2∶suc2Δ* cells increases because the cells that cannot make invertase are too far from those that can. But at high densities, *suc2Δ* cells outcompete *SUC2* cells, presumably because they do not have to bear the expense of producing invertase [24].

In well-stirred environments, much of the monosaccharides produced by invertase escape into the bulk medium, suggesting that *suc2Δ* cells would fare well even at low cell densities. This prediction is valid for single cells, but if cells grow as clumps, the cells in the *SUC2* clumps should cooperate to capture a higher fraction of the monosaccharides they produce and thus have an increased advantage over the cells that cannot produce invertase. The same reasoning applies to the initial stages of growth in cultures that are not stirred: the *SUC2* clumps will start dividing well before single *SUC2* cells and will thus have a greater advantage over *suc2Δ* cells.

**Figure 5:**
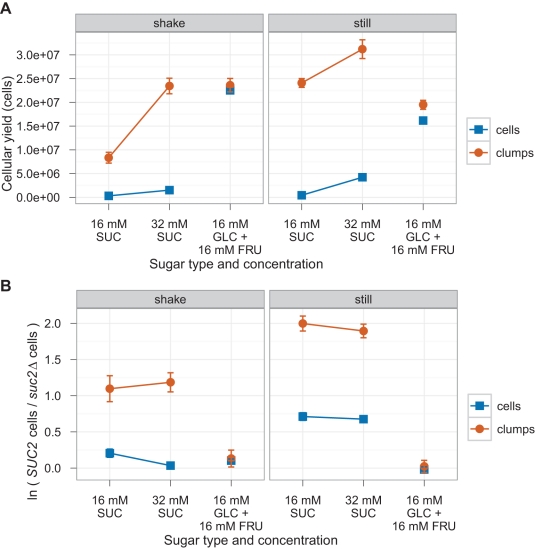
Competition between invertase producers (*SUC2*) and non-producers (*suc2*Δ). 60 *SUC2* cells and 60 *suc2Δ* cells or three 15–25 cell *SUC2* clumps and three 15–25 cell *suc2Δ* clumps were sorted into 150 µl wells at the given sugar concentration and allowed to grow for 72 h at 30°C. During incubation, the 96-well plates were either shaken at 1,000 RPM (left column) or left still (right column). (A) Cellular yield of *SUC2* strains in competition with *suc2*Δ strains. Optical density (OD) values were converted to cell population by measuring the optical density of dilutions of a culture with known cell densities. (B) The growth advantage of various *SUC2* strains over a *suc2Δ* strain. ln( *SUC2* cells/*suc2Δ* cells) is proportional to the difference in the mean growth rate, assuming both strains start with an equal number of cells. The population frequencies were counted using a FACS machine: in half the samples *SUC2* strains expressed mCitrine from the *ACT1* promoter and *suc2*Δ strains expressed mCherry from the *ACT1* promoter, and in the other half of the samples, the colors were reversed. Single cells expressed the *amn1-W303* allele and clumps expressed the *AMN1-RM11* allele. *SUC2* strains yJHK401 (cell) and yJHK390 (clump) express mCitrine, and *SUC2* strains yJHK410 (cell) and yJHK391 (clump) express mCherry. *suc2*Δ strains yJHK302 (cell) and yJHK433 (clump) express mCitrine, and *suc2*Δ strains yJHK437 (cell) and yJHK435 (clump) express mCherry. 24 samples were counted in each color combination. Error bars refer to the 95% confidence interval calculated using the one-sample Student's *t* test.

We tested these predictions by mixing *SUC2* and *suc2Δ* strains and following their growth in low sucrose concentrations while we manipulated two variables: whether the cells were growing as clumps or as single cells, and whether the cultures were shaken or not. Microtiter wells were inoculated with either 60 single cells (*amn1-W303*) or three 15–25 cell clumps (*AMN1-RM11*) of each of the two genotypes (*SUC2* or *suc2Δ*), and either shaken or held still at 30°C for 72 h. We measured two outcomes: the overall cellular yield (Figure 5A) and the logarithm of the ratio of *SUC2* to *suc2Δ* cells (Figure 5B).

The data show that clumps of *SUC2* cells have two advantages over the corresponding number of *SUC2* single cells: they produce higher numbers of cells and they fare better in the competition against “cheats” that cannot make invertase. These conclusions hold at two sucrose concentrations (16 and 32 mM) and for both still and shaken cultures.

## Discussion

In the evolution from unicellularity to multicellularity, the clustering of individual cells into a multicellular clump of undifferentiated cells was a necessary precursor to all subsequent innovations such as division of labor and germ-soma separation. Selection can only favor clumps if the fitness of an average cell in a clump exceeds the fitness of an average cell that is not part of a clump. Many selective advantages of multicellular clumps have been proposed; most fall into one of two categories: protection or nutrient usage. Clumping has been shown to provide protection from phagocytosis [32], multicellular predators [33], and environmental stresses [34]. Colony formation has also been proposed as a means of protection [35]. Dworkin proposed that a high cell density was required for myxobacteria to hydrolyze insoluble nutrients, creating an advantage for the swarming behavior of myxobacteria [36]. And Pfeiffer and Bonhoeffer, using a computer simulation, proposed that clustering could have allowed more efficient energy usage by reducing interactions with cheats [37]. Because multicellularity evolved independently multiple times [2], it is possible that different mechanisms accounted for different origins. We propose that incomplete cell separation gave cells an advantage in both the use of growth-promoting secretions and exclusion of cheaters.

Our data link a simple social trait, the use of secreted products, with a simple form of multicellularity, incomplete separation after cell division. Making small clumps of cells allows yeast strains to more effectively use invertase to break down sucrose: clumps of cells grow at sucrose concentrations where an equivalent number of single cells cannot (Figure 4). This advantage comes from at least two mechanisms. First, the diffusion of monosaccharides from all cells in the clump raises the concentration of monosaccharides at the cell membrane available for import and growth (Figure 3). Second, the higher level of glucose in the clump should stimulate the higher expression of invertase in the clump (Figure S1A), creating a positive feedback loop. Because the local cell density alters the concentration of a molecule (glucose) that regulates gene expression, this positive feedback is a primitive form of quorum sensing.

The clumps we have studied contain modest numbers of cells. Thus even if a cheat arises by mutation during the growth of a clump, fragmentation of the clump and further cell proliferation will rapidly produce clumps that are composed entirely of either cooperators (invertase producers) or cheats [38]. The fact that all cells in a clump share a single recent ancestor means that effective kin selection can occur (note that lineage-independent forms of aggregation do not guarantee kin selection). In both still and shaken medium, the multicellular clumps perform better than single cells when in the presence of a “cheater” *suc2Δ* strain that can use the sugars hydrolyzed by *SUC2* cells without itself contributing to the hydrolysis. This advantage is stronger in shaken media, where there is the strongest potential for *suc2Δ* cells to exploit secretor strains (Figure 5).

Although the genetics and physiology of budding yeast have been well characterized, our knowledge of yeast ecology is modest [39]. *S. cerevisiae* has been found in a wide variety of environments such as damaged grapes in Italy [40], rotting figs in California [10], soil near oak trees in Pennsylvania [41], prickly pear in the Bahamas, White teff in Ethiopia, and Bertram Palm nectar in Malaysia [42]. How budding yeast disperse is unknown. Insects are one potential vector and budding yeast have been found in *Drosophila* [43] and *Vespa crabro* (D. Cavalieri, personal communication). Dispersal by insects is consistent with the idea that cells will be widely spread and face growth from low densities, which would select against *suc2Δ* strains.

We speculate that the formation of cell clumps by incomplete cell separation arose after the use of secreted products. In unstirred environments, the repeated division of a single cell will produce a high local density of genetically identical cells, even if cell separation is complete, ensuring that secreted products tend to benefit the same genotypes [37],[44]. The widespread occurrence of secreted enzymes among diverse prokaryotes and unicellular eukaryotes suggests that cooperation among cells evolved long before the multicellularity of eukaryotes. But once cells used secreted products, incomplete separation would allow genetically identical cells to cooperate with each other in stirred as well as unstirred environments. The benefits of sharing the products of hydrolytic enzymes could have selected for undifferentiated multicellularity.

## Materials and Methods

### Media and Strains

All synthetic media used in this research were prepared immediately before the assay from refrigerated 10× yeast nitrogen base (YNB), refrigerated sugar stock, and filtered water. No amino acids or nucleotides were added; all strains used in this research were prototrophic (Table S4). The YNB was based on the recipe of Wickerham [45], with the following modifications: first, riboflavin and folic acid were not added to the YNB to minimize autofluorescence [46]; and second, inositol was not added to the YNB in order to eliminate a potential carbon source. YP 2% glycerol was made with 10 g/l yeast extract, 20 g/l peptone, and 2% (v/v) glycerol. YEP was made with 10 g/l yeast extract and 20 g/l peptone. See Table S2 for the YNB recipe. Unless otherwise noted, all chemicals used in this research were purchased from Sigma-Aldrich (http://www.sigmaaldrich.com/).

### FACS-Inoculated Plate Assays

Cells were pregrown in 1 mM glucose media for at least 12 h to ensure the cells were expressing invertase prior to inoculation. Cells undergoing galactose induction in Figure S6 were diluted and grown an additional 8 h in 1 mM glucose plus 1 mM galactose media. Cells undergoing galactose induction in Figure S7 were pregrown in YP 2% glycerol plus the indicated concentration of galactose. The cells were inoculated into 96-well plates using a MoFlo FACS (Beckman Coulter, http://www.beckmancoulter.com/) with 3 excitation lasers: 440 nm, 488 nm, and 594 nm. Gating for single cells and clumps was done on a pulse width versus fluorescence plot. The fluorescence channel was chosen to correspond to the constitutively produced fluorescent protein: 488 nm laser with 550/30 nm filter for *P_ACT1_*-ymCitrine, and 594 nm laser with 630/40 nm filter for *P_ACT1_*-ymCherry. Before inoculation, 10 cells and 10 clumps were spotted on a microscope slide and checked under a microscope to verify the gate was properly set for a clump size of one cell (a single unbudded cell or cell plus its bud), 15–30 cells (Figures 4, S6, and S7), or 15–25 cells (Figure 5). After inoculation, the plates were covered with foil to prevent evaporation and incubated at 30°C.

Plates were analyzed using a Typhoon (GE Healthcare Life Sciences. http://www.gelifesciences.com/) laser scanner at 50 µm resolution, +3 µm focal plane, 488 nm laser, 520/40 emission filter, and 500 V photomultiplier tube (PMT). Wells with growth were manually counted from the Typhoon images and checked by visually inspecting the plates. Population ratios in the *SUC2/suc2Δ* competitions (Figure 5) were measured using a BD LSRFortessa cell analyzer (BD Biosciences, http://www.bdbiosciences.com/); 70 µl of each sample was measured to a maximum of 100,000 cells. The FACS files were analyzed using FlowJo Flow Cytometry Analysis Software (FlowJo, http://www.flowjo.com/).

Optical densities were measured on a Spectramax Plus 384 (Molecular Devices, http://www.moleculardevices.com/) absorbance microplate reader. OD595 values were converted to cell density by measuring dilutions of a culture whose density was measured using a Coulter Counter (Beckman Coulter, http://www.beckmancoulter.com/). Non-clumpy (*amn1-W303*) cells were sonicated prior to Coulter measurement. Clumpy (*AMN1-RM11*) cells were not sonicated; instead, the number of cells in the culture was found by multiplying the number of Coulter counts by 6.4, which is the average clump size calculated by visually counting 388 clumps using a confocal microscope. Separate calibration curves were made for cells and for clumps.

### Data Analysis and Figures

Data analysis was performed using custom-written scripts in the R programming language (http://www.r-project.org/). The Adjusted Wald method of calculating 95% binomial confidence intervals [47] was used because a low number (<100) of samples were used to generate a binomial mean. Plots were generated using ggplot2 (http://had.co.nz/ggplot2/). Figures were prepared using OmniGraffle (The Omni Group, http://www.omnigroup.com/) and Adobe Illustrator (www.adobe.com). The image in Figure 1B was generated using MATLAB (www.mathworks.com). The following abbreviations are used in the figures: FRU, fructose; GAL, galactose; GLC, glucose; SUC, sucrose.

### Simulation

Parameters and the algorithm used in the simulation are detailed in Table S2.

## Supporting Information

Figure S1 Invertase expression and activity. (A) Activity of the *SUC2* promoter as a function of extracellular glucose concentration for cells grown in minimal synthetic media. FACS was used to measure the fluorescent intensity of mCherry driven by the *SUC2* promoter, which was normalized using a constitutively expressed mCitrine driven by the *ACT1* promoter (strain yJHK383). The length of the error bar corresponds to one standard deviation. Cells were grown in the given concentration of glucose in exponential phase for 12 h before measurement. (B) Internal and external enzyme activities of the prototrophic *SUC2* strain yJHK222 and prototrophic *suc2-1cyt* strain yJHK290, measured as the number of molecules of glucose liberated per second in 128 mM sucrose in pH = 4.5 tartrate buffer. Cultures were washed and inoculated from an exponentially growing culture into the specified concentration of glucose plus YEP at ≈1E5 cell/ml and grown for 6 h. The cultures were then washed and resuspended in 1 mM potassium phosphate, pH = 7.5, at 1.5E7 cell/ml. The cultures were split into two: one for intact cell invertase activity, and one for lysed cell invertase activity. 0.5% Zymolyase (Zymo Research Corp, http://www.zymoresearch.com/) was added to each of the lysed cell cultures. The cells were incubated at 30°C for 45 min to allow lysis to occur. 1.5E5 (10 µl) cells or cell equivalent were then added to prewarmed 390 ml 5 mM tartrate buffer (pH = 4.5). 100 µl of prewarmed 640 mM sucrose was added and sucrose hydrolysis was allowed to occur at 30°C for 35 min. Samples were then diluted 10∶1 in 50 mM sodium phosphate (pH = 7.5) plus 0.25 mM N-ethylmaleimide [18]. The amount of glucose in each sample was then measured using an Amplex Red Glucose Assay Kit (Invitrogen, http://www.invitrogen.com/). The external invertase activity data points correspond to the mean intact cell measurements and the internal activity data points correspond to the mean lysed cell measurements minus the mean intact cell measurements. Three technical replicates were performed per sample. The error bars in the external activity measurements refer to the 95% confidence interval calculated using the one-sample Student's *t*-test of the three replicates, and the error bars in the internal activity measurements refer to the 95% confidence interval calculated using the two-sample Student's *t* test of the three replicates (external and lysed activity). *suc2Δ* strain yJHK302 was also measured in parallel and used as a zero reference. *suc2-1cyt* strain yJHK290 was measured at ½ mM glucose and 16 mM glucose only. (C) Michaelis-Menten curve of invertase activity for the prototrophic *SUC2* strain yJHK222. Cells were pregrown in 0.5 mM glucose and inoculated into various levels of sucrose and incubated as described above (without the cell lysis step) for 28 min to determine the rate of sucrose hydrolysis by invertase. Four samples were used per data point; error bars refer to the one-sample Student's *t* test. The R function nls (nonlinear least squares) was used to fit the shown Michaelis-Menten curve to the data set and to obtain the following values: K_m_ = 11 mM sucrose, V_max_ = 3.6E8 molecule glucose s^−1^ cell^−1^. *suc2Δ* strain yJHK302 was also measured in parallel and used as a zero reference. (D) Growth rate in YEP plus various concentrations of glucose of the prototrophic strain yJHK222. Cultures were inoculated from an exponentially growing culture into the specified concentration of glucose plus YEP at 2,000 cell/ml. Cultures were first grown for 8 h, and then samples were taken at four time points over the next 6 h. Samples were briefly sonicated and then measured using a Coulter Counter (Beckman Coulter, http://www.beckmancoulter.com/). Three replicates were measured in parallel for each glucose concentration. The R function nls (nonlinear least squares) was used to find an exponential growth rate for each set of four time points. The error bar for each data point on the plot refers to 95% confidence interval for the three replicates.
(EPS)

Figure S2 Lab yeast strains cannot grow at low sucrose concentrations. Cells were inoculated by FACS into 150 µl wells at the given cell density and sugar concentration and allowed to grow for 85 h without shaking at 30°C. The fraction of wells with growth using S288C background strain yJHK361 is shown (this figure is similar to Figure 1 except the strain is S288C background instead of W303 background). Results shown are totals of three experiments; each experiment used one plate for each sugar concentration / strain combination. Error bars refer to 95% binomial confidence interval using the adjusted Wald method.
(EPS)

Figure S3 Typical fluorescent scan of a plate containing 16 mM sucrose inoculated with the indicated number of cells. Note the faint and uniform growth in the wells containing 256 and 512 cells. If only a small fraction of cells were capable of growing in low concentrations of sucrose, we would expect to see a few discrete colonies at the highest cell numbers, rather than the nearly uniform growth that we observe. The contrast of this image was increased to improve visibility.
(EPS)

Figure S4 Simulation of glucose uptake in isolated cells and a cell clump. The simulated local glucose concentration and glucose uptake of a cell inoculated at the center of a 150 µl sphere in two environments: at the center of a clump of 30 cells and at the center of a total of 30 cells uniformly dispersed throughout the volume. (A) 2 mM sucrose: glucose intake rate of the cell as a function of time after inoculation. (B) 32 mM sucrose: glucose intake rate of the cell as a function of time after inoculation. Note the logarithmic scale on the *y*-axis. See Supporting Information for details of code and parameters.
(EPS)

Figure S5 Simulation of glucose uptake at the center of different sizes of cell clump. The simulated local glucose concentration and glucose uptake of a cell inoculated at the center of a 150 µl sphere. (A) Glucose concentration as a function of radial distance from the center of the cell after 8 h of incubation. Note the logarithmic scale on the *x*-axis. (B) Glucose intake rate of the cell as a function of time after inoculation. Note the logarithmic scale on the *y*-axis. Cells continue to consume low levels of glucose at large clump size because sucrose diffuses into the clump and is available for immediate hydrolysis and consumption. See Supporting Information for details of code and parameters.
(EPS)

Figure S6 Clumps of cells produced by a variety of methods have a growth advantage over an equal number of single cells in low sucrose concentrations. Cells were inoculated by FACS as described in Figure 4. (Top) Galactose-induced *AMN1-RM11* strains yJHK226 (*SUC2*) and yJHK227 (*suc2Δ*). Cells were pregrown without galactose to produce single cells or with galactose to produce clumps. The assay medium contained sucrose but lacked galactose. (Bottom) Galactose-induced *CTS1* (chitinase) strains yJHK228 (*SUC2*) and yJHK229 (*suc2Δ*). Cells were pregrown with galactose to produce single cells or without galactose to produce clumps. The assay medium contained sucrose but lacked galactose. Results shown are totals of three experiments; each experiment used one plate for each sugar concentration / clumpiness-induction-method combination, and each plate represents 24 wells for each combination of genotype and clumpiness. Error bars refer to 95% binomial confidence interval using adjusted Wald method.
(EPS)

Figure S7 Clumps of cells have a growth advantage over an equal number of single cells when *SUC2* is expressed constitutively. 30 cells or a single 15–30 cell clump were inoculated by FACS into 150 µl wells at the given sugar and galactose concentration and grown for 85 h at 30°C without shaking. In the invertase-producing, *AMN1-RM11* strain yJHK315, *SUC2* is driven by the *GAL1* promoter (*P_GAL1_-SUC2*). *SUC2* is deleted in the *suc2Δ* strain yJHK317. Galactokinase (*GAL1*) is deleted from both strains so that galactose acts as an inducer and not as a carbon source. Results shown are totals of three experiments; each experiment used one plate for each sugar concentration / induction-level combination, and each plate represents 24 wells for each combination of genotype and clumpiness. Error bars refer to 95% binomial confidence interval using adjusted Wald method. Galactose was added to all wells in the following concentrations: (Top) 1/16 mM, (Middle) 1/8 mM, and (Bottom) 3/16 mM.
(EPS)

Table S1 Parameters used for software simulation and description of algorithm.
(DOC)

Table S2 Yeast nitrogen base recipe.
(DOC)

Table S3 Fitness cost of endogenous invertase expression for exponentially growing cells.
(DOC)

Table S4 Yeast strains.
(DOC)

## Abbreviations

FACS: fluorescence activated cell sorter
OD: optical density
PMT: photomultiplier tube
YNB: yeast nitrogen base
