# Correction: Sucrose Utilization in Budding Yeast as a Model for the Origin of Undifferentiated Multicellularity

**DOI:** 10.1371/annotation/0b9bab0d-1d20-46ad-b318-d2229cde0f6f

**Published:** 2011-08-29

**Authors:** John H. Koschwanez, Kevin R. Foster, Andrew W. Murray

There were errors in author names in the citation. The correct citation is: Koschwanez JH, Foster KR, Murray AW (2011) Sucrose Utilization in Budding Yeast as a Model for the Origin of Undifferentiated Multicellularity. PLoS Biol 9(8): e1001122. doi:10.1371/journal.pbio.1001122 

