# Supplementary material for: Sucrose Utilization in Budding Yeast as a Model for the Origin of Undifferentiated Multicellularity
Source: PLoS Biol. 2011 Aug 9;9(8):e1001122. doi: 10.1371/journal.pbio.1001122 (PMC3153487; doi:10.1371/journal.pbio.1001122)
Supplement: Table S1 — Parameters used for software simulation and description of algorithm. (DOC) [file pbio.1001122.s008.doc]

**Table S1: Parameters used for software simulation and description of algorithm**

| Parameter | Value | Reference |
| --- | --- | --- |
| Glucose diffusion coefficient in water (and cell wall and cell clump) | 670 µm^2^/s |  |
| Sucrose diffusion coefficient in water (and cell wall and cell clump) | 520 µm^2^/s |  |
| Yeast cell volume | 42 µm^3^ |  |
| Yeast cell radius | 2.2 µm | derived from cell volume |
| Cell wall thickness | 0.2 µm |  |
| Outer boundary | 3.3 mm | derived from sphere of 150 µl (inoculation volume) |
| Glucose transport K_m_ | 0.8 mM (high affinity)  21 mM (low affinity) |  |
| Glucose transport V_max_ | 167 nmol min^-1^ mg^-1^ (high affinity)  104 nmol min^-1^ mg^-1^ (low affinity) |  |
| Cell dry weight | 15E-12 g |  |
| Activity of purified invertase octamer | 3E4 molecule glucose s^-1^ |  |
| Invertase K_m_ | 11 mM sucrose | Figure S1C |
| Invertase production per cell in equivalent purified octamers per second | | Glucose concentration | Equivalent purified invertase production (molecule/s) | | --- | --- | | 0 | 0.46 | | 0.125 | 0.55 | | 0.25 | 0.61 | | 0.50 | 0.50 | | 1.0 | 0.31 | | 2.0 | 0.21 | | 4.0 | 0.15 | | 8.0 | 0.10 | | >16 | 0 | | Figures S1B, S1D, and activity of purified invertase octamer |

The diffusion simulation was written in the Python programming language (http://www.python.org/). The basic algorithm is as follows:

1. Set up a one-dimensional radial grid with the central cell at the center of the grid and with the wall boundary at the outer boundary of the grid. The innermost grid point is the cell membrane. Four different sub-grids of composite media are used: the cell wall, the clump, a fine-grained region of media and a course-grained region of media.
2. Set up a diffusion matrix for three components: glucose, sucrose, and invertase. Glucose and invertase have flux boundaries at the cell membrane, and sucrose has a reflective boundary at the cell membrane (flux = 0). All three components have reflective boundaries at the outer wall. Each component’s flux across each internal boundary between composite media is continuous. The initial glucose concentration and invertase concentrations are zero, and the initial sucrose concentration is set to the same value at all points.
3. Perform the following actions at each one-second time point:
   1. From the glucose concentration at the cell membrane, set the outward flux of invertase and the inward flux of glucose at the inner boundary (cell membrane). Set the glucose consumed and the invertase produced by the clump. It is assumed that there is no time delay between invertase production and secretion to the cell wall.
   2. Allow the sucrose, glucose, and invertase to diffuse.
   3. Record the concentrations of each diffusing molecule and the glucose flux of the center membrane.

Diffusion is solved using the Crank-Nicholson method for solving partial differential equations . The linsolve function from the SciPy (http://www.scipy.org/) library was used to solve the matrices. Python and SciPy were both used as part of the Enthought Python Distribution (http://www.enthought.com).

.

**Table S1 References**

1. Lide DR (2008) CRC Handbook of Chemistry and Physics: CRC Press.

2. Jorgensen P, Edgington NP, Schneider BL, Rupes I, Tyers M, et al. (2007) The size of the nucleus increases as yeast cells grow. Mol Biol Cell 18: 3523-3532.

3. Schmidt M, Strenk ME, Boyer MP, Fritsch BJ (2005) Importance of cell wall mannoproteins for septum formation in Saccharomyces cerevisiae. Yeast 22: 715-723.

4. Reifenberger E, Boles E, Ciriacy M (1997) Kinetic characterization of individual hexose transporters of Saccharomyces cerevisiae and their relation to the triggering mechanisms of glucose repression. Eur J Biochem 245: 324-333.

5. Sherman F (2002) Getting started with yeast. Meth Enzymol 350: 3-41.

6. Esmon PC, Esmon BE, Schauer IE, Taylor A, Schekman R (1987) Structure, assembly, and secretion of octameric invertase. J Biol Chem 262: 4387-4394.

7. Crank J (1980) The Mathematics of Diffusion: Oxford University Press, USA. 424 p.
