# Supplementary material for: Sucrose Utilization in Budding Yeast as a Model for the Origin of Undifferentiated Multicellularity
Source: PLoS Biol. 2011 Aug 9;9(8):e1001122. doi: 10.1371/journal.pbio.1001122 (PMC3153487; doi:10.1371/journal.pbio.1001122)
Supplement: Table S2 — Yeast nitrogen base recipe. (DOC) [file pbio.1001122.s009.doc]

Table S2. Yeast nitrogen base recipe

| **Chemical** | **1X concentration (mg/l)** |
| --- | --- |
| Ammonium sulfate | 5000 |
| Magnesium sulfate | 500 |
| Sodium chloride | 100 |
| Calcium chloride | 100 |
| Potassium phosphate monobasic | 1000 |
| Boric acid | 0.5 |
| Copper (II) sulfate | 0.04 |
| Potassium iodide | 0.1 |
| Iron (III) chloride | 0.2 |
| Manganese sulfate | 0.4 |
| Sodium molybdate | 0.2 |
| Zinc sulfate | 0.4 |
| Biotin | 0.002 |
| Calcium pantothenate | 0.4 |
| niacin | 0.4 |
| PABA | 0.2 |
| Pyridoxine HCl | 0.4 |
| Thiamine hydrochloride | 0.4 |
