# Supplementary material for: Sucrose Utilization in Budding Yeast as a Model for the Origin of Undifferentiated Multicellularity
Source: PLoS Biol. 2011 Aug 9;9(8):e1001122. doi: 10.1371/journal.pbio.1001122 (PMC3153487; doi:10.1371/journal.pbio.1001122)
Supplement: Table S3 — Fitness cost of endogenous invertase expression for exponentially growing cells. (DOC) [file pbio.1001122.s010.doc]

**Table S3** **Fitness cost of endogenous invertase expression for exponentially growing cells.**

| Competition | s: 80 mM glucose: | s: 1 mM glucose: | **change in s from 80 mM to 1 mM glucose** |
| --- | --- | --- | --- |
| *suc2∆* mCitrine (1)  *suc2∆* mCherry (2) | -0.00056  (-0.0020, 0.00091) | 0.00006  (-0.0032, 0.0033) | **0.00062**  (-0.0021, 0.0033) |
| *SUC2* mCitrine (1)  *suc2*∆ mCherry (2) | -0.0029  (-0.0047, -0.0011) | -0.0066  (-0.0088 ,-0.0044) | **-0.0037**  (-0.0056, -0.0019) |
| *SUC2* mCherry (1)  *suc2*∆ mCitrine (2) | -0.0019  (-0.0027, -0.0011) | -0.0052  (-0.0075, -0.0030) | **-0.0033**  (-0.0053, -0.0011) |

To measure fitness cost of endogenous invertase, each strain was individually inoculated into liquid YPD directly from frozen glycerol stock. The strains were grown to saturation and then diluted into the test media and grown for an additional 12 hours, not allowing cells to reach saturation. At least 100,000 cells of each strain were then mixed in 30 ml of the test media: YEP plus the given concentrations of glucose. The 30 ml culture was then split between 3 glass tubes for the 3 technical replicates. These tubes were then grown at 30 ºC in a rotating drum for 12 hours, not allowing cells to reach saturation, in preparation for time point “t0”. Every 12 hours for four total time points, cell concentration of one replicate in each competition was measured using a Coulter Counter (Beckman Coulter, http://www.beckmancoulter.com/). 200,000 cells from each replicate were then diluted into a prewarmed glass tube containing identical media and placed back into the rotating drum at 30 ºC. Cultures were maintained at a concentration below saturation (<1E7 cells/ml in 80 mM glucose and <1.5E6 cells/ml in 1 mM glucose). At least 150,000 cells from each replicate were also analyzed on a MoFlo FACS (Beckman Coulter, http://www.beckmancoulter.com/) in order to measure the population ratio.

The FACS files were analyzed using FlowJo Flow Cytometry Analysis Software (FlowJo, http://www.flowjo.com/) in order to find the number of cells in each population. The fitness value was obtained by analyzing the populations using custom-written software in the R programming language. The analysis steps were as follows:

1. Find the log ratio of the populations for each replicate at each time point.
2. Find the number of generations at each time point for one of the two strains.
3. Fit a line to log ratio vs. time in generations. The slope of this line is the value of *s*.
4. Find the mean value of s and the 95% confidence interval (using the 1-sample t-test) among the three technical replicates.
5. In comparing two media conditions, find the mean difference and the 95% confidence interval using the 2-sample t-test.

All values of s refer to the selective advantage of strain 1 (listed first with a (1)) over strain 2 (listed second with a (2)). The values of s listed in parentheses refer to the 95% confidence interval calculated using 1- or 2-sample Student’s t-test, as appropriate for the three replicates in each competition.

Strains (all are prototrophic and express identical drug markers):

*SUC2* mCitrine: yJHK401

*SUC2* mCherry: yJHK410

*suc2*∆ mCitrine: yJHK302

*suc2*∆ mCherry: yJHK437
