# Supplementary material for: Sucrose Utilization in Budding Yeast as a Model for the Origin of Undifferentiated Multicellularity
Source: PLoS Biol. 2011 Aug 9;9(8):e1001122. doi: 10.1371/journal.pbio.1001122 (PMC3153487; doi:10.1371/journal.pbio.1001122)
Supplement: Table S4 — Yeast strains. (DOC) [file pbio.1001122.s011.doc]

**Table S4**: Yeast strains

| Strain | Background strain | *AMN1* allele | Constitutive color marker | *SUC2* changes | *MAL* changes | Additional changes |
| --- | --- | --- | --- | --- | --- | --- |
| yJHK111 | W303 *BUD4* ; MATa ;  *can1-100* | *amn1-W303* | *his3*∆::*P_ACT1_-*ymCitrine-*tADH1-His3MX6* |  |  |  |
| yJHK112 | W303 *BUD4* ; MATa ;  *can1-100* | *amn1-W303* | *his3*∆::*P_ACT1_-*ymCherry-*tADH1-His3MX6* |  |  |  |
| yJHK222 | W303 *BUD4* ; MATa ;  *can1-100* | *amn1-W303* | *his3*∆::*P_ACT1_-*ymCitrine-*tADH1-His3MX6* |  | *mal11/12*∆::*hphMX4* |  |
| yJHK223 | W303 *BUD4* ; MATa ;  *can1-100* | *AMN1-RM11* | *his3*∆::*P_ACT1_-*ymCitrine-*tADH1-His3MX6* |  | *mal11/12*∆::*hphMX4* |  |
| yJHK224 | W303 *BUD4* ; MATa ;  *can1-100* | *AMN1-RM11* | *his3*∆::*P_ACT1_-*ymCitrine-*tADH1-His3MX6* | *suc2∆*::*natMX4* | *mal11/12*∆::*hphMX4* |  |
| yJHK226 | W303 *BUD4* ; MATa ;  *can1-100 ;*  *his3-11,15* | *AMN1-RM11* | *ura3∆*::*P_ACT1_-*ymCitrine-*tADH1-URA3* |  | *mal11/12∆*::*ble* | *gal1/10∆*::*hphMX4;*  *P_GAL3_*∆::*His3MX6- P_ACT1_-GAL3;*  *P_AMN1_∆*::*kanMX6-* *P_GAL1_-AMN1-RM11* |
| yJHK227 | W303 *BUD4* ; MATa ;  *can1-100 ;*  *his3-11,15* | *AMN1-RM11* | *ura3∆*::*P_ACT1_-*ymCitrine-*tADH1-URA3* | *suc2∆*::*natMX4* | *mal11/12∆*::*ble* | *gal1/10∆*::*hphMX4;*  *P_GAL3_∆*::*His3MX6- P_ACT1_-GAL3;*  *P_AMN1_∆*::*kanMX6- P_GAL1_-AMN1-RM11* |
| yJHK228 | W303 *BUD4* ; MATa ;  *can1-100 ;*  *his3-11,15* | *AMN1-RM11* | *ura3∆*::*P_ACT1_-*ymCitrine-*tADH1-URA3* |  | *mal11/12∆*::*ble* | *gal1/10∆*::*hphMX4;*  *P_GAL3_∆*::*His3MX6- P_ACT1_-GAL3;*  *P_CTS1_∆*::*kanMX6- P_GAL1_-CTS1* |
| yJHK229 | W303 *BUD4* ; MATa ;  *can1-100 ;*  *his3-11,15* | *AMN1-RM11* | *ura3∆*:: *P_ACT1_-*ymCitrine-*tADH1-URA3* | *suc2∆*::*natMX4* | *mal11/12∆*::*ble* | *gal1/10∆*::*hphMX4;*  *P_GAL3_∆*::*His3MX6- P_ACT1_-GAL3;*  *P_CTS1_∆*::*kanMX6- P_GAL1_-CTS1* |
| yJHK259 | W303 *BUD4* ; MATa ;  *can1-100* | *AMN1-RM11* | *his3*∆::*P_ACT1_-*ymCitrine-*tADH1-His3MX6* | *suc2-1cyt* | *mal11/12*∆::*hphMX4* |  |
| yJHK290 | W303 *BUD4* ; MATa ;  *can1-100* | *amn1-W303* | *his3*∆::*P_ACT1_-*ymCitrine-*tADH1-His3MX6* | *suc2-1cyt* | *mal11/12*∆::*hphMX4* |  |
| yJHK302 | W303 *BUD4* ; MATa ;  *can1-100* | *amn1-W303* | *his3*∆::*P_ACT1_-*ymCitrine-*tADH1-His3MX6* | *suc2*∆::*kanMX6* | *mal11/12*∆::*hphMX4* |  |
| yJHK315 | W303 *BUD4* ; MATa *;*  *can1-100 ;*  *his3-11,15* | *AMN1-RM11* | *ura3∆*::*P_ACT1_-*ymCitrine-*tADH1-URA3* | *P_SUC2_*∆::*kanMX6- P_GAL1_-SUC2* | *mal11/12*∆::*hphMX4* | *gal1/10∆*::*LEU2*;  *P_GAL3_∆*::*His3MX6- P_ACT1_-GAL3* |
| yJHK317 | W303 *BUD4* ; MATa *;*  *can1-100 ;*  *his3-11,15* | *AMN1-RM11* | *ura3∆*::*P_ACT1_-*ymCitrine-*tADH1-URA3* | *suc2∆*::*kanMX6* | *mal11/12*∆::*hphMX4* | *gal1/10∆*::*LEU2*;  *P_GAL3_∆*::*His3MX6- P_ACT1_-GAL3* |
| yJHK361 | S288C (BY4714) | *amn1-W303* | *his3*∆::*P_ACT1_-*ymCitrine-*tADH1-His3MX6* |  |  |  |
| yJHK383 | W303 *BUD4* ; MATa ;  *can1-100* | *amn1-W303* | *his3*∆::*P_ACT1_-*ymCitrine-*tADH1-His3MX6* | *P_SUC2_-ymCherry-tSUC2-NatMX4-P_SUC2_-SUC2* | *mal11/12*∆::*hphMX4* |  |
| yJHK390 | W303 *BUD4* ; MATa ;  *can1-100* | *AMN1-RM11* | *his3*∆::*P_ACT1_-*ymCitrine-*tADH1-His3MX6* |  | *mal11/12*∆::*hphMX4* | *ho∆*::*kanMX4* |
| yJHK391 | W303 *BUD4* ; MATa ;  *can1-100* | *AMN1-RM11* | *his3*∆::*P_ACT1_-*ymCherry-*tADH1-His3MX6* |  | *mal11/12*∆::*hphMX4* | *ho∆*::*kanMX4* |
| yJHK401 | W303 *BUD4* ; MATa ;  *can1-100* | *amn1-W303* | *his3*∆::*P_ACT1_-*ymCitrine-*tADH1-His3MX6* |  | *mal11/12*∆::*hphMX4* | *ho∆*::*kanMX4* |
| yJHK410 | W303 *BUD4* ; MATa ;  *can1-100* | *amn1-W303* | *his3*∆::*P_ACT1_-*ymCherry-*tADH1-His3MX6* |  | *mal11/12*∆::*hphMX4* | *ho∆*::*kanMX4* |
| yJHK433 | W303 *BUD4* ; MATa ;  *can1-100* | *AMN1-RM11* | *his3*∆::*P_ACT1_-*ymCitrine-*tADH1-His3MX6* | *suc2∆*::*kanMX6* | *mal11/12*∆::*hphMX4* |  |
| yJHK435 | W303 *BUD4* ; MATa ;  *can1-100* | *AMN1-RM11* | *his3*∆::*P_ACT1_-*ymCherry-*tADH1-His3MX6* | *suc2∆*::*kanMX6* | *mal11/12*∆::*hphMX4* |  |
| yJHK437 | W303 *BUD4* ; MATa ;  *can1-100* | *amn1-W303* | *his3*∆::*P_ACT1_-*ymCherry-*tADH1-His3MX6* | *suc2∆*::*kanMX6* | *mal11/12*∆::*hphMX4* |  |

**Strain notes**:

1. All strains are *MATa* and prototrophic, and were created for this project. All strains except for yJHK361 are from a W303 background and contain the S288C (corrected) allele of *BUD4* (standard W303 strains have a mutation in *BUD4* [1].) *BUD4* was corrected by the authors by using plasmid pJHK047.

2. Strain yJHK361 is derived from BY4714 (S288C background), which was a generous gift from the Boeke lab [2].

3. ymCherry is a yeast optimized version of mCherry [3] and was generously provided by Nicolas Ingolia of the Weissman Lab. ymCitrine is a yeast optimized version of mCitrine [4].

4. In the galactose-induction strains, *GAL3* was placed on the *ACT1* promoter in order to achieve a graded response from galactose [5].

5. *HO* was deleted by *kanMX4* (*ho∆::kanMX4*) in some strains in order to match drug markers with other strains used in the same experiment. All strains used in this study are heterothallic.

6. *amn1-W303* was replaced by *AMN1-RM11* by using plasmid pEF607, which was a generous gift from the Kruglyak Lab [6].

7. *His3MX6*, *kanMX6* (G418 resistance), *natMX4* (Clonnat resistance), and *hphMX4* (hygromycin resistance) come from pFA6a-series plasmids [7,8].

8. *ble* (phleomycin resistance) comes from plasmid pUG66 [9].

9. *MAL11* and *MAL12* are not active in lab yeast strains [10] and were deleted in all but two strains to match strains used in other ongoing and unpublished research by the authors.

10. We produced a form of the *SUC2* gene that could only produce cytoplasmic invertase (see figure S1B). We created the *suc2-cyt1* allele by deleting the two ATG codons that precede the transmembrane domain of the secreted form of Suc2. This allele is similar to other alleles that have been shown to only produce cytoplasmic invertase [11,12].

11. To request strains or plasmids, please see instructions on the Murray Lab web site (http://www.mcb.harvard.edu/murray/contact.html).

**Table S4 references:**

1. Voth WP, Olsen AE, Sbia M, Freedman KH, Stillman DJ (2005) ACE2, CBK1, and BUD4 in budding and cell separation. Eukaryotic Cell 4: 1018-1028.

2. Brachmann CB, Davies A, Cost GJ, Caputo E, Li J, et al. (1998) Designer deletion strains derived from Saccharomyces cerevisiae S288C: a useful set of strains and plasmids for PCR-mediated gene disruption and other applications. Yeast 14: 115-132.

3. Shaner NC, Campbell RE, Steinbach PA, Giepmans BNG, Palmer AE, et al. (2004) Improved monomeric red, orange and yellow fluorescent proteins derived from Discosoma sp. red fluorescent protein. Nat Biotechnol 22: 1567-1572.

4. Sheff MA, Thorn KS (2004) Optimized cassettes for fluorescent protein tagging in Saccharomyces cerevisiae. Yeast 21: 661-670.

5. Ingolia NT, Murray AW (2007) Positive-feedback loops as a flexible biological module. Curr Biol 17: 668-677.

6. Yvert G, Brem RB, Whittle J, Akey JM, Foss E, et al. (2003) Trans-acting regulatory variation in Saccharomyces cerevisiae and the role of transcription factors. Nat Genet 35: 57-64.

7. Longtine MS, McKenzie A, Demarini DJ, Shah NG, Wach A, et al. (1998) Additional modules for versatile and economical PCR-based gene deletion and modification in Saccharomyces cerevisiae. Yeast 14: 953-961.

8. Goldstein AL, McCusker JH (1999) Three new dominant drug resistance cassettes for gene disruption in Saccharomyces cerevisiae. Yeast 15: 1541-1553.

9. Gueldener U, Heinisch J, Koehler GJ, Voss D, Hegemann JH (2002) A second set of loxP marker cassettes for Cre-mediated multiple gene knockouts in budding yeast. Nucleic Acids Research 30: e23.

10. Brown CA, Murray AW, Verstrepen KJ (2010) Rapid expansion and functional divergence of subtelomeric gene families in yeasts. Curr Biol 20: 895-903.

11. Kaiser CA, Botstein D (1986) Secretion-defective mutations in the signal sequence for Saccharomyces cerevisiae invertase. Mol Cell Biol 6: 2382-2391.

12. Perlman D, Raney P, Halvorson HO (1986) Mutations affecting the signal sequence alter synthesis and secretion of yeast invertase. Proc Natl Acad Sci USA 83: 5033-5037.
